# Location Matters: Navigating Regional Heterogeneity of the Neurovascular Unit

**DOI:** 10.3389/fncel.2021.696540

**Published:** 2021-06-30

**Authors:** Louis-Philippe Bernier, Clément Brunner, Azzurra Cottarelli, Matilde Balbi

**Affiliations:** ^1^Djavad Mowafaghian Centre for Brain Health, University of British Columbia, Vancouver, BC, Canada; ^2^Neuro-Electronics Research Flanders, Leuven, Belgium; ^3^Vlaams Instituut voor Biotechnologie, Leuven, Belgium; ^4^Interuniversity Microeletronics Centre, Leuven, Belgium; ^5^Department of Neurosciences, KU Leuven, Leuven, Belgium; ^6^Irving Medical Center, Columbia University, New York, NY, United States; ^7^Queensland Brain Institute, University of Queensland, Brisbane, QLD, Australia

**Keywords:** neurovascular unit, regional heterogeneity, blood-brain barrier, pericytes, astrocytes, endothelial cell

## Abstract

The neurovascular unit (NVU) of the brain is composed of multiple cell types that act synergistically to modify blood flow to locally match the energy demand of neural activity, as well as to maintain the integrity of the blood-brain barrier (BBB). It is becoming increasingly recognized that the functional specialization, as well as the cellular composition of the NVU varies spatially. This heterogeneity is encountered as variations in vascular and perivascular cells along the arteriole-capillary-venule axis, as well as through differences in NVU composition throughout anatomical regions of the brain. Given the wide variations in metabolic demands between brain regions, especially those of gray vs. white matter, the spatial heterogeneity of the NVU is critical to brain function. Here we review recent evidence demonstrating regional specialization of the NVU between brain regions, by focusing on the heterogeneity of its individual cellular components and briefly discussing novel approaches to investigate NVU diversity.

## Introduction

The varied and complex tasks performed by the brain have energy demands that far outweigh those of other organs. Accordingly, the vascular system that supplies the brain has evolved into an intricate arrangement that is unique in its cellular composition and function. The neurovascular unit (NVU) is comprised of a diverse population of cells including neurons, endothelial cells (ECs), pericytes, astrocytes, vascular smooth muscle cells (vSMCs) and others, that act in a coordinated manner to spatially and temporally match the local blood supply to the energy demand of neural activity (Iadecola, 2017). Together, NVU cells form the blood brain barrier (BBB), a regulated semipermeable border unique to the brain that shields the parenchyma from the vessel lumen (Sweeney et al., 2019).

**Figure 1:**
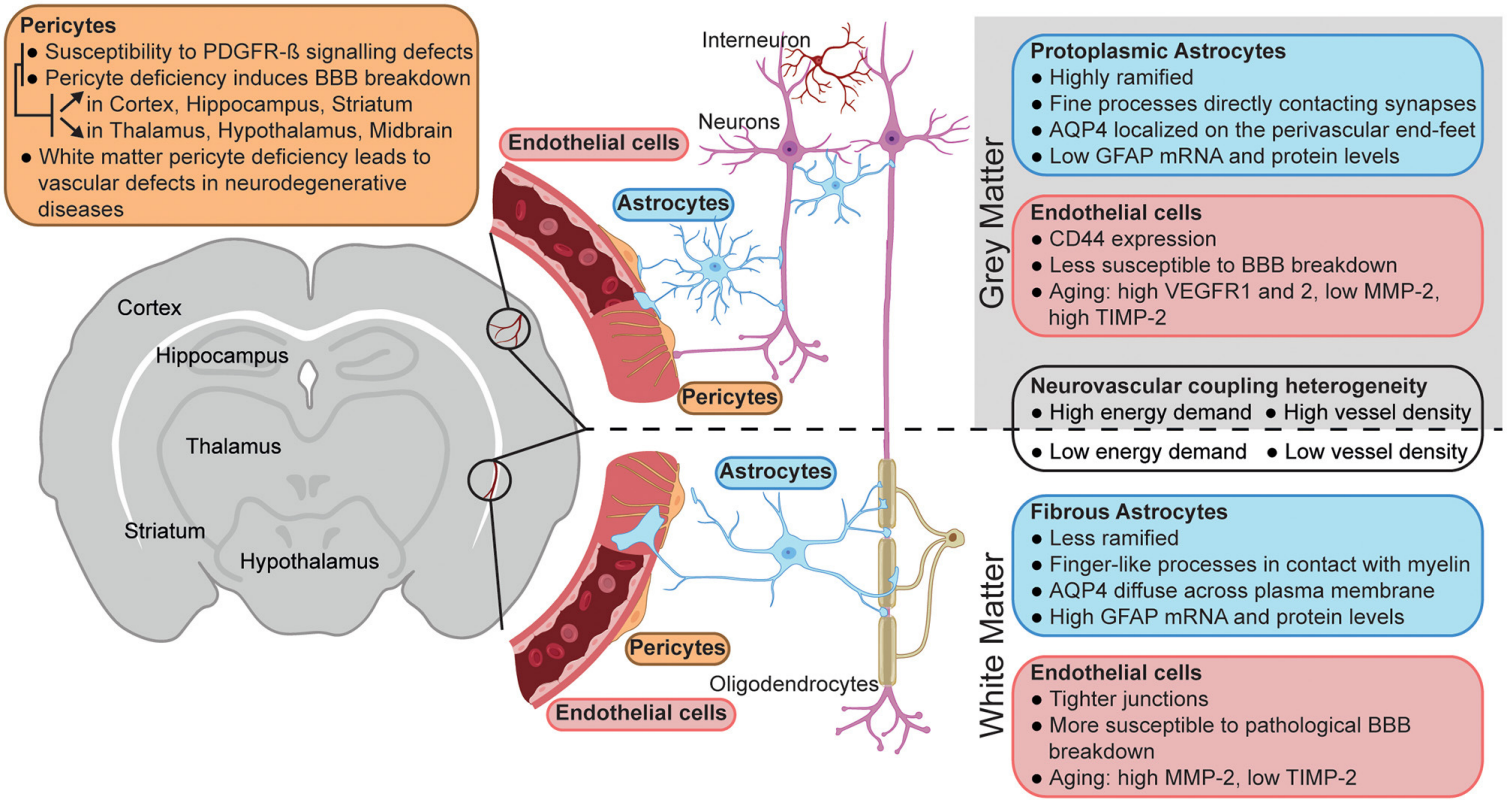
Neurovascular unit heterogeneity between gray and white matter. Schematic representation of the main differences between the neurovascular unit of gray (top) vs. white matter (bottom). The figure was created using BioRender.com.

The complexity of neurovascular communications has been recognized for a long time; however, new tools have recently allowed us to distinguish the various cell populations of the NVU in greater detail. We now know that the NVU not only harbors diverse cell populations, but that these cell populations are regionally heterogeneous. This regional heterogeneity can be broadly divided into large and local scales. On a large scale, this review will highlight recent evidence showing that NVU cells, and the communication between them, vary depending on their location within specific anatomical or functional brain region. On a more local scale, spatial NVU cellular heterogeneity is also found along the vascular tree [see reviews (Wilhelm et al., 2016; Noumbissi et al., 2018; Villabona-Rueda et al., 2019)]. Recent demonstrations of such vessel type-dependent variability include gene expression analysis of ECs that line the vessel lumen, which suggests that BBB function differs with vascular tree location (Vanlandewijck et al., 2018). Abluminal to the ECs, distinct populations of mural cells exist from arteriolar smooth muscle cells to capillary pericytes. Different phenotypes of pericytes, whether defined by morphology or molecular identity, exist along the arterio-venular tree (Hartmann et al., 2015; Vanlandewijck et al., 2018). Likewise, astrocytic gene expression and end-foot coverage is partially governed by vessel type and size (Wang M. X. et al., 2021). New studies have also uncovered previously unrecognized roles for perivascular fibroblasts and perivascular macrophages, preferentially found in the arteriolar and venular compartments (Vanlandewijck et al., 2018; Koizumi et al., 2019). It is therefore becoming increasingly recognized that spatial heterogeneity of NVU components along this arteriole-capillary-venule axis is necessary to allow local neurovascular coupling (NVC) and functional hyperemia to take place. While this vessel type-dependent heterogeneity has been reviewed recently (Wilhelm et al., 2016; Noumbissi et al., 2018; Villabona-Rueda et al., 2019), NVU function also displays heterogeneity on a wider scale, where the vascular compartment needs to match the varying demands of distinct anatomical regions of the brain. Here, we review recent evidence showing that this regional variability in neurovascular communication relies on the individual cellular components of the NVU differing between the functional and anatomical regions of the brain (Figure 1) and briefly discuss new tools available to probe this spatial heterogeneity.

## Brain Region-Specific Neurovascular Coupling in Health and Disease

The brain is separated into anatomical regions that are interconnected yet functionally distinct. The relative specialization of each region allows the CNS to execute incredibly complex tasks, and their varying functions require adapted blood flow control. Furthermore, a widely different activity profile and cellular composition exists between synapse-rich gray matter areas and white matter harboring myelinated axonal fibers. More importantly for neurovascular activity, the corresponding energy demand of these diverse regions varies extensively. Glucose consumption is 2–4 times greater in gray matter (Sokoloff et al., 1977) where major energy-consuming processes like synaptic activity and action potential generation take place (Attwell and Laughlin, 2001). In white matter, resting potential maintenance and housekeeping functions in both neurons and oligodendrocytes are believed to require a comparatively lower metabolic demand (Harris and Attwell, 2012). The differential energy demands of gray matter and white matter translate into adapted microvascular structural architecture: vascular density is higher in gray matter, with arterioles and venules preferentially oriented perpendicular to cortical layers while vessels in white matter are organized parallel to axonal fibers (Cavaglia et al., 2001; Hase et al., 2019). This suggests that the coupling between local neural activity and blood flow needs to vary regionally, and recent data shows that a varying cellular composition of the NVU may underlie this adaptation.

Adding to evidence of regional heterogeneity of NVU activity under physiological conditions is the region-dependent neurovascular dysfunction observed in the pathogenesis of various brain diseases. It is now widely recognized that vascular abnormalities are key pathogenic features of cognitive decline. A group of disorders broadly termed vascular cognitive impairment and dementias (VCID) is thought to be responsible for ~20% of dementias (Iadecola, 2013; Gorelick et al., 2016; Kim et al., 2020). Cerebral small vessel disease (CSVD)—a disease of varying etiology that affects small arterioles and venules, and deep capillaries of the brain—underlies a majority of VCID cases. It results in impaired NVC, reduced blood flow, and increased BBB permeability. Neuroimaging characteristics of CSVD include subcortical infarcts and white matter hyperintensities, implying a regional component of CSVD pathogenesis (Shi and Wardlaw, 2016; Cuadrado-Godia et al., 2018). White matter abnormalities linked to cerebrovascular dysfunction are also a risk factor for Alzheimer's disease (AD) (Alber et al., 2019). The links between dementias and cerebrovascular dysfunction, specifically in white matter, have brought attention to the differential composition of the NVU between gray matter and white matter and the differential effects of NVU dysfunction on neural health. Taken together, the communication between the vascular system and neural parenchyma shows region-specific variability in health and disease, drawing interest to potential region-specific properties of the individual cell types making up the NVU.

## Endothelial Cells

ECs lining CNS vessels form the first line of defense against circulating toxins and pathogens, while bearing responsibility for the entrance of indispensable nutrients and ions. This extremely selective control of permeability is achieved via three distinct features: (1) specialized tight junctions restricting paracellular flow, (2) low rates of transcytosis hampering transcellular transport, and (3) expression of membrane transporters mediating the influx of nutrients and the efflux of toxic waste (Biswas et al., 2020).

While endothelial heterogeneity along the arterio-venous tree has been extensively described (Sabbagh et al., 2018; Vanlandewijck et al., 2018), inter-regional differences in endothelial properties are subtle and mostly manifested in pathological conditions. BBB integrity is tightly linked to the expression pattern of junctional proteins in ECs and to restriction of transcytotic pathways and minor differences have been identified in the BBB signature of ECs in gray matter compared to white matter. In particular, the expression of junctional proteins occludin, claudin-5 and α-catenin is higher in white matter ECs compared to cortical ECs. Functionally, this reflects *in vitro* into a higher transendothelial electrical resistance of primary microvascular cells isolated from the white matter compared to those isolated from the cortex (Nyul-Toth et al., 2016). Although ECs in the white matter seemingly form a tighter paracellular barrier, white matter vessels are reported to be more susceptible than gray matter vessels to junctional fragmentation and loss of occludin and ZO-1 in pathological conditions such as HIV-associated neurocognitive deficits (Dallasta et al., 1999). Consistently with these observations, several groups have reported that white matter vasculature is more susceptible to pathological hyperpermeability. In multiple sclerosis (MS) patients, local BBB dysfunction predominantly affects white matter but not gray matter (Taheri et al., 2011). Additionally, several cognitive impairments, including VCID, AD and post-stroke dementia display marked BBB permeability specifically in the white matter, likely as a response to a marked loss of pericytes in those regions (Hase et al., 2018; Ding et al., 2020).

Regional variability of BBB-forming ECs is somewhat more prominent in the circumventricular organs (CVOs), where an active exchange between blood and the CNS is required. Compared to ECs elsewhere in the brain, ECs in CVOs have a lower expression of junctional proteins, higher rates of transcytosis and higher expression of the fenestration-forming plasmalemma vesicle-associated protein, resulting in increased permeability to tracers (Morita et al., 2016). This distinctively different signature of CVOs ECs may be due to low canonical Wnt signaling, as activation of Wnt/β-catenin pathway, by stabilizing β-catenin in ECs, induces a BBB-like phenotype (Benz et al., 2019; Wang et al., 2019). Similar to CVOs, a permeable vasculature is necessary in the choroid plexus (ChP), the site of cerebrospinal fluid (CSF) secretion. ECs in the ChP are fenestrated to allow the passage of water and small molecules. The blood-CSF barrier at the ChP is instead formed by a layer of epithelial cells surrounding choroidal vessels that regulate the permeability by expressing junctional proteins and membrane transporters (Dani et al., 2021).

Aging has very distinct and regional-specific effects on CNS vasculature. As both vascular density and branching decrease across most brain regions with a more pronounced vessel loss in white matter as a result of aging (Murugesan et al., 2012; Schager and Brown, 2020), ECs in white and gray matter activate specific pro-angiogenic pathways to maintain vessel health. In gray matter, ECs start expressing higher levels of vascular endothelial growth factor receptor-1 (VEGFR1) and VEGFR2, while Angiopoietin-1 (Ang1) is specifically upregulated in the cortex. In white matter, ECs upregulate the expression of the matrix metalloproteinase-2 (MMP-2) and downregulate its inhibitor TIMP-2; conversely, ECs in the cortex express less MMP-2 and more TIMP-2 during aging (Murugesan et al., 2012). While minimal decrease in vessel branching is observed in the hippocampus compared to cortex and white matter, it is the first brain area to show age-dependent increase in BBB permeability (Montagne et al., 2015), possibly as a response to the increase of Ang2 that destabilizes the vasculature (Murugesan et al., 2012). Studies focused specifically on hippocampal vasculature have shown a prominent switch from ligand-dependent transport to caveolar transport across the BBB in aged mice, together with a marked decrease in junctional and matrix organization (Yang et al., 2020).

Some regional heterogeneity in the expression of transporters and adhesion molecules on the EC membrane also exists. The expression of the efflux transporter P-glycoprotein (P-gp) was found to be higher in the cortex than in the cerebellum (Yasuda et al., 2015). Additionally, in epileptogenic tissue resected from the temporal lobe, P-gp expression was higher in gray matter capillaries, and its expression in both white and gray matter correlated with increased seizure recurrence (Kwan et al., 2010). Given the key role of P-gp in preventing drugs from entering the CNS parenchyma, identifying the variability in its distribution at the BBB is fundamental to address the mechanisms of pharmacoresistance (Liu et al., 2015). The expression of the adhesion molecule CD44 is restricted to ECs within gray matter (Kaaijk et al., 1997). This region-specific pattern is relevant in the context of neuroinflammation, as CD44 mediates the interaction of ECs with immune cells and its deficiency increases BBB disruption in an experimental autoimmune encephalitis (EAE) model (Flynn et al., 2013).

Aside from the features outlined in this review, the regional heterogeneity of ECs is still largely unknown. A deeper investigation in this variability is fundamental when modeling vascular pathology *in vitro*, since the area and method of isolation of ECs from the brain may influence their phenotype and behavior in culture.

## Mural Cells

Mural cells are critical players in the control of local blood flow. They line most vessels of the brain and include vSMCs and pericytes. vSMCs are arranged in circumferential bands wrapping around arterioles where they are key mediators of vascular contractility and in a mesh-like pattern on venules. Pericytes line capillaries and post-capillary venules and are embedded within the basement membrane with a characteristic bump-on-a-log cell body. Pericytes are morphologically heterogeneous, with a cell shape highly correlated to its relative location along the arteriole-capillary-venule axis. “Ensheating pericytes” expressing contractile machinery populate 1st–4th-order branches, “thin-strand pericytes” are located along deeper capillaries and “mesh pericytes” are found on post-capillary venules. Pericyte heterogeneity is such that identification of putative subpopulations requires a combination of location, morphology, marker expression, and function. The nomenclature and the defining molecular markers attributed to each type of pericyte, and the presence of discrete subpopulations vs. a gradual continuum of phenotypes remains hotly debated (Hartmann et al., 2015; Hill et al., 2015; Attwell et al., 2016; Vanlandewijck et al., 2018). While mural cell populations along the arterial-venous tree are locally diverse, recent studies also hint at a functional diversity of mural cells, most notably pericytes, on a larger scale with brain region-dependent characteristics (Hartmann et al., 2015; Cudmore et al., 2017; Nikolakopoulou et al., 2017; Villasenor et al., 2017).

Pericytes are found lining vessels in all brain regions; however, the density of platelet-derived growth factor receptor-β (PDGFR-β)-expressing pericytes in the cortex inversely correlates with neural cell density, such that neuron-rich layers II/III of the cortex contain 40% less pericytes than layer I (Hartmann et al., 2015). Although vascular density itself may differ between cortical layers, this observation was corroborated by a study detailing a decreased density of CD13^+^ pericytes per capillary length in layer II/III (Cudmore et al., 2017). With pericyte presence often linked to tonic vessel constriction (Fernandez-Klett et al., 2010; Hall et al., 2014; Gonzales et al., 2020; Hartmann et al., 2021), it is suggested that a higher pericyte density may act as a buffer to blood flow pressures in upper cortical layers while lower pericyte density in cell body-rich layers may contribute to vessel relaxation necessary for NVC in areas with higher metabolic demand. A recent whole-brain analysis of pericyte density showed elevated density of PDGFRβ-expressing pericytes in deeper cortical layers (layers IV and V) relative to layer I, with pericyte density generally matching vascular density across most regions (Wu et al., 2021). Notable exceptions were thalamic areas, where a relatively higher pericyte density (relative to local vascular density) was observed.

In addition to their role in the control of vascular tone, pericytes participate in angiogenesis and vessel stabilization, as well as in the maturation and maintenance of BBB integrity. Interestingly, this key role of pericytes in BBB maintenance was shown to vary substantially among anatomical brain regions. Signaling through PDGFR-β on pericytes is key for pericyte recruitment to vessels and BBB generation and maintenance (Armulik et al., 2010; Daneman et al., 2010). Disruption of the PDGF-B/PDGFR-β signaling axis induces pericyte loss, disruption of pericyte-endothelial communication and subsequent vascular defects and BBB dysfunction (Armulik et al., 2010; Bell et al., 2010; Daneman et al., 2010; Mae et al., 2021). This property has been investigated by various groups to demonstrate region-specific contributions of pericytes to BBB function. In one model where genetic ablation of the retention motif of PDGF-B prevents pericyte recruitment (*pdgf-b*^*ret*/*ret*^) (Armulik et al., 2010), the subsequent BBB leakage is elevated in the cortex, hippocampus and striatum compared to deeper areas such as the interbrain and midbrain (Villasenor et al., 2017). The regional variability in extravasation of Evans Blue and Gd-DTPA and parenchymal IgG accumulation was observed in *pdgf-b*^*ret*/*ret*^ mice despite a uniform reduction of CD13^+^ pericyte coverage throughout those same brain regions (~75% less than in controls). This suggests that the relative importance of pericytes in maintaining BBB integrity varies among brain regions, beyond their local susceptibility to PDGFR-β signaling defects. In another model of pericyte deficiency, where 7 point mutations on the *pdgfrb* gene (*pdgfrb*^*F*7/*F*7^) (Tallquist et al., 2003) prevents normal endothelial-pericyte signaling, regional differences in the BBB dependence on pericyte presence was confirmed. The progressive loss of CD13^+^ pericytes induced elevated BBB breakdown in the cortex, hippocampus and striatum as compared to thalamus (Nikolakopoulou et al., 2017). In this transgenic model, as well as in a pericyte-specific ablation model based on double expression of PDGFR-β and NG2 (Nikolakopoulou et al., 2019), BBB dysfunction directly correlated with regional pericyte loss. Taken together, the vulnerability of NVU function to pericyte loss is region-dependent, and the general resilience of thalamic areas to pericyte dysfunction may be linked to the elevated pericyte:vessel ratio these regions harbor (Wu et al., 2021).

Nevertheless, pericyte vulnerability shows a component of regional variability, which may play an important role in several pathologies. Vascular defects in white matter of the brain, and pericyte dysfunction, are believed to be partially responsible for multiple types of dementias (Sagare et al., 2013; Halliday et al., 2016; Alber et al., 2019; Nortley et al., 2019; Ding et al., 2020). In *pdgfrb*^*F*7/*F*7^ mice, pericyte loss was prominently observed in white matter tracts, such as corpus callosum, in association with BBB leakage and accumulation of blood-borne fibrinogen (Montagne et al., 2018). Such deposits of fibrinogen are thought to be toxic to oligodendrocytes, inducing demyelination and white matter damage. In multiple sclerosis (MS), the nature of the associated neurological deficits are highly dependent on the location of focal inflammatory immune cell infiltration and ensuing neural tissue lesions (Alvarez et al., 2015). When EAE, a mouse model of MS, was combined with the *pdgf-b*^*ret*/*ret*^ model of pericyte deficiency, the regional immune infiltration correlated with regional pericyte loss (Torok et al., 2021). The local reduction in pericyte coverage drives an increased VCAM-1 and ICAM-1 expression on ECs, thereby facilitating trafficking of pro-inflammatory CD45^+^ leukocytes into the brain. Since vascular defects and BBB dysfunction are hallmarks of neurodegenerative diseases, this highlights how a regional variation in pericyte susceptibility to disturbances in PDGFR-β signaling, or other yet unrecognized pathways, may in fact underlie the regional component of those diseases.

The mechanisms that dictate pericyte heterogeneity across brain regions are still poorly understood, but regionally specialized functions may drive a certain pericyte phenotype. For example, in the paraventricular nucleus, where vessel density is 3–5-fold higher than in the cortex, pericyte coverage of vessels is also significantly higher (Frahm and Tobet, 2015). In CVOs, where the BBB lacks tight junctions to allow sensing of blood-borne cues such as osmolarity, pericytes display especially high levels of NG2 and PDGFR-β (Morita et al., 2014). These levels were increased upon chronic osmotic stimulation induced via salt-loading, unlike in the cortex where they remained unchanged. Interestingly, this challenge also promoted BBB leakage in CVOs. Ontogenic variability may also play a role in the observed heterogeneity of pericytes among anatomical brain regions. Indeed, the developmental origin of brain pericytes is heterogeneous, with studies demonstrating neural crest lineage, mesenchymal lineage and hematopoietic sources (Etchevers et al., 2001; Trost et al., 2016; Yamamoto et al., 2017; Yamazaki and Mukouyama, 2018). The ontogeny of pericytes in specific brain regions remains to be elucidated but is likely to participate in the regional heterogeneity of pericyte function.

## Astrocytes

Maintenance of the BBB is not a function of endothelial and mural cells alone, but a dynamic process that involves parenchymal cells including astrocytes, which express a specific molecular repertoire essential for the physiology of the vascular system.

Astrocytes are a heterogeneous subtype of glial cells that play key roles in BBB formation and upkeep, blood flow regulation and vascular contractility in response to neural activity (Abbott et al., 2010; Alvarez et al., 2013; Boulay et al., 2016). They release glia-derived neurotrophic factors and provide metabolic support to neurons (Sofroniew and Vinters, 2010; Allaman et al., 2011; Allen and Eroglu, 2017). Astrocytes display both inter- and intra-regional differences in their morphology, gene expression, and physiological role. Differences in astrocytic distribution and morphology between white and gray matter reflect the distinct functions of these CNS components and meet the specific functional requirements that each astrocytic population must fulfill. In white matter, oligodendrocytes create a myelin sheath around axonal fibers (Marques et al., 2016) that restricts direct contact of fibrous astrocytes with axonal fibers to gaps in this sheath called nodes of Ranvier (Black and Waxman, 1988; Lubetzki et al., 2020). These fibrous astrocytes are small with few but elongated, finger-like branches and organized in rows around white matter tracts (Miller, 2018; Matias et al., 2019). Conversely, gray matter—or protoplasmic—astrocytes have fine processes that envelope ~200,000 synapses in mice, and ~2 million in humans (Oberheim et al., 2006). Protoplasmic astrocytes are highly ramified cells, with greater process arborization in layers II/III than in layer IV (Lanjakornsiripan et al., 2018). Patterns of gene expression specific to gray matter astrocytes differ between cortical layers and areas (Morel et al., 2019; Batiuk et al., 2020; Bayraktar et al., 2020), suggesting a high degree of intra-regional heterogeneity in the mouse brain. Importantly, astrocytes not only contact neuronal elements, but also directly interact with the vasculature via specialized end-feet.

A critical role of astrocytes is to maintain the barrier properties of endothelial cells forming the BBB. Astrocyte cell bodies are on average located 6–10 μm from blood vessels (McCaslin et al., 2011), and their end-feet form a continuous sheath around all vessels below the pia including arterioles, capillaries, and venules (McCaslin et al., 2011). Astrocytic end-feet cover 90–99% of the brain's vasculature (Abbott et al., 2006; Mathiisen et al., 2010; Lundgaard et al., 2014). This astrocytic extension to the vessels' wall forms a subcellular domain dedicated to the gliovascular interaction with abundant aquaporin-4 (AQP4), potassium channels (KIR4.1), glucose transporter 1 (GLUT-1/SLC2A1), and connexin-43 (Abbott et al., 2006; Jessen et al., 2015), highlighting the astrocytes' role in regulating water transport as well as blood and glymphatic flow—the product of the exchange of the brain interstitial and cerebrospinal fluid mediated by AQP4 and responsible for the brain's waste clearance via the glymphatic pathway (Iliff and Simon, 2019; Rasmussen et al., 2021). Along the vascular tree, the perivascular astrocytic sheath in the somatosensory cortex is thicker in arterioles than in capillaries (Jammalamadaka et al., 2015). However, capillaries have the greatest density of astrocyte end-foot processes, and the separation between astrocytes and capillaries decreases with increasing cortical depth (McCaslin et al., 2011).

Inter-regional differences in astrocytes can be observed in their protein expression (Morel et al., 2017). A prime example of regional differences is AQP4, a channel protein selectively permeable to water that is highly expressed in astrocytic membranes at the BBB and blood-CSF barrier, indicating its role in controlling bidirectional fluid exchange (Papadopoulos and Verkman, 2013). Interestingly, *in vivo* AQP4 deletion did not affect BBB permeability to macromolecules, with no changes in glial fibrillary acidic protein (GFAP) expression, or microvessels ECs morphology (Saadoun et al., 2009; Haj-Yasein et al., 2011). However, the consequent uncoupling of water and potassium transport can determine susceptibility to other conditions since a decrease in AQP4 leads to an increase in propensity for seizures and cognitive decline (Yang et al., 2011) and participates in pathologies associated to brain edema (Bonomini and Rezzani, 2010; Stokum et al., 2015). While the distribution pattern of AQP4 mRNA is homogeneous across the cortex and white matter, the protein itself is expressed in the white matter at almost half of the level detected in the cortex (Nyul-Toth et al., 2016)—likely due to the lower levels of vessel density that characterize the white matter. AQP4 in protoplasmic astrocytes is preferentially localized to the perivascular end-feet, while fibrous astrocytes show a more uniform localization across the plasma membrane (Stokum et al., 2015). This difference in distribution of one of the main regulators of water homeostasis could explain why white matter is more vulnerable to swelling and edema under ischemic conditions.

Astrocytes are highly coupled via gap junctions (Cotrina and Nedergaard, 2012) comprised of connexins Cx30 and Cx43, which allow astrocytes to exchange ions and molecules below 1.5 kDa with adjacent cells by connecting their cytoplasm (Bruzzone et al., 1996). Double deletion of Cx30 and Cx43 downregulates AQP4 (Nielsen et al., 1997) and weakens the BBB, which becomes permeable to macromolecules upon an increase in vascular pressure (Ezan et al., 2012). Interestingly, the expression of these connexins is not uniform across anatomical circuits: corpus callosum astrocytes are less coupled to each other by gap junctions than their counterparts in the neocortex (Haas et al., 2006), showing that gap junctional communication between astrocytes can differ among brain regions. Whether these regional differences in connexin expression directly affect communication between astrocytes and ECs remains to be clarified.

Astrocytes express high levels of GFAP, which is also upregulated in reactive astrocytes and is often associated with the severity of CNS disorders (Escartin et al., 2019), though this correlation may be impacted by the regional differences in basal GFAP expression (Griemsmann et al., 2015; Ben Haim and Rowitch, 2017). For instance, hippocampal astrocytes display a higher level of GFAP than cortical, thalamic, or striatal astrocytes (Bushong et al., 2002; Chai et al., 2017). GFAP expression is also higher in astrocytes isolated from the corpus callosum compared to those in cortical structures (Goursaud et al., 2009). In fact, mRNA and proteins levels of GFAP are 3–7-fold higher in white matter than in gray matter (Nyul-Toth et al., 2016). Although direct investigations in the mechanistic role of GFAP in NVU function remain limited, its region-dependent expression pattern is involved in white matter vs. gray matter vascular properties since GFAP deletion increases the permeability to macromolecules of the BBB in white matter and specifically reduces vascularization in white matter compared to gray matter (Liedtke et al., 1996).

Regional identities of astrocytes are maintained over time, and evidence has shown that aged astrocytes from different brain regions present a unique molecular signature (Boisvert et al., 2018; Clarke et al., 2018). Significant differences in age-related changes in gene expression have been found between hippocampus, striatum and cortex, with an up-regulation of astrocytic reactive genes in the hippocampus and striatum—regions that show more susceptibility to oxidative stress and other environmental stressors (Saxena and Caroni, 2011)—compared to the cortex (Clarke et al., 2018). Accordingly, RNA sequencing studies on aging astrocytes from different regions showed that cortical astrocytes undergo minimal changes in gene expression (<100 differentially regulated genes) compared to cerebellar astrocytes (>500 genes). Interestingly, an age-dependent increase in GFAP expression has been shown in the hippocampus, but also in the frontal and temporal cortices (Boisvert et al., 2018). However, functional implication of GFAP upregulation during aging and CNS disorders is still controversial. A failure of astrocytes to adequately adapt to their roles in areas affected by aging may lead to a loss of neuronal populations due to insufficient vascularization, and differences in the regional expression of key proteins may be crucial to our understanding of the origin and progression of neurodegenerative diseases.

## Tools to Study Regional Heterogeneity of the NVU

Investigations in the NVU diversity demonstrate region-dependent cellular variations, however direct functional investigations remain necessary to evaluate how these differences in NVU composition affect local brain functions. In this regard, the development of novel neuroimaging modalities suitable for brain-wide recordings has expanded the toolset required to probe the regional heterogeneity of the NVU that was until recently mostly addressed by single cell RNA sequencing (scRNAseq) and functional magnetic resonance imaging (fMRI). The former is a major contributor in characterizing the single cell transcriptomic profile of the cellular and vascular components of the NVU highlighting region-specific differences at the brain-wide scale (Saunders et al., 2018; Ross et al., 2020) and beyond what can be achieved with conventional imaging modalities. The latter, fMRI, is a gold standard modality suitable to capture the heterogeneity of hemodynamic responses at the whole brain scale (Devonshire et al., 2012) and between white and gray matter (Gawryluk et al., 2014). However, when considering NVU/C investigations scRNAseq lacks the functional interplay between cellular and vascular components while fMRI approach remains limited by a reduced spatiotemporal resolution when compared with other modalities (Urban et al., 2017), the need of anesthetic drugs when considering their impact on the NVC (Aksenov et al., 2015), and its lack of cellular component compensated by complementary modalities (Mishra et al., 2011; Aksenov et al., 2015).

**Figure 2:**
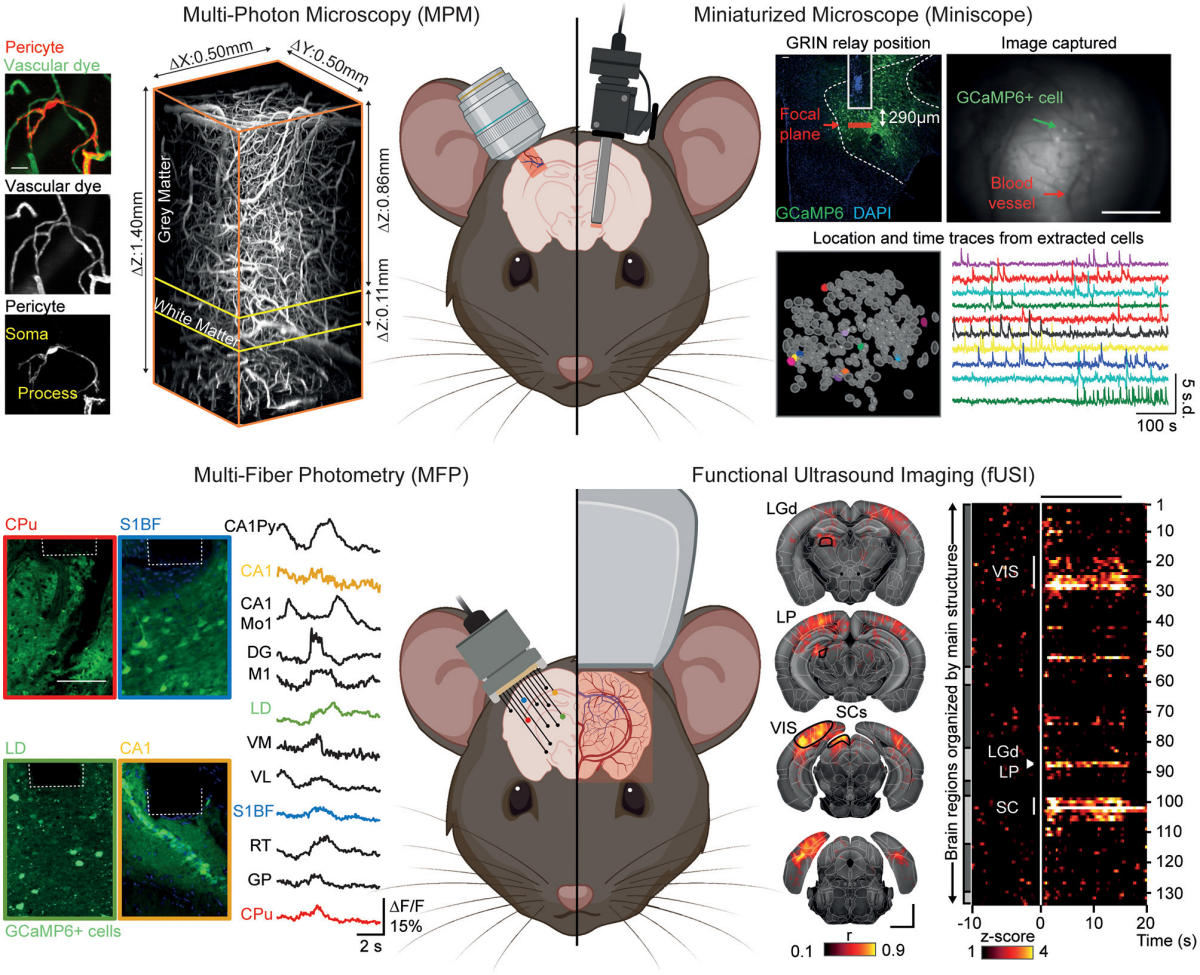
Neuroimaging modalities to investigate the regional heterogeneity of the neurovascular unit under awake conditions. Top left: Multi-photon microscopy (MPM) allows high-resolution imaging of cell-specific activities (here, pericytes) and vascular dynamics using fluorescent dyes in a cortical column and deeper regions. Top right: Miniaturized microscope (Miniscope) suitable for imaging of single cell activity and blood vessels from deep brain regions (here, lateral hypothalamus) by the mean of a GRIN relay. Bottom left: Multi-fiber photometry (MFP) allows for simultaneous recording of cellular changes (here, GCaMP6m-expressing cells) from across, but not limited to, 12 individual brain regions. Bottom right: Functional ultrasound imaging (fUSI) allows for high sensitivity recording of hemodynamic changes with a brain-wide coverage of more than 130 regions per hemisphere. Reprinted with permission from Li B. et al. (2020), Berthiaume et al. (2018), Resendez et al. (2016), Sych et al. (2019), and Brunner et al. (2020). The figure was created using BioRender.com.

Hereafter we focus on recent strategies allowing for cellular and/or vascular imaging under conscious conditions enabling to preserve the physiological integrity of the NVC (Masamoto and Kanno, 2012; Reimann and Niendorf, 2020). While acting at different spatiotemporal scales, multi-photon microscopy (MPM; 2- and 3-photon microscopy), miniaturized fluorescence microscopes (Miniscope), multi-fiber photometry (MFP), and functional ultrasound imaging (fUSI) modalities are complementary methods providing information on the interplay between the cellular and vascular components of the NVU regionally and at the larger, whole brain scale (Figure 2). For further comparison of neuroimaging methods described in this section, we also refer the readers to Urban et al. (2017) and Walter et al. (2020).

Multi-photon microscopy (MPM) is the “gold standard” for the study of cellular dynamics, function and morphology as well as hemodynamics (Shih et al., 2012; O'Herron et al., 2016; Urban et al., 2017) in a low- to non-invasive way [i.e., cranial window (Goldey et al., 2014), thinned-skull (Drew et al., 2010), or transcranial (Wang et al., 2018)]. The capabilities of MPM (Urban et al., 2017; Li B. et al., 2020) combined with cell-specific genetically encoded activity reporters suited for probing either calcium or voltage changes (GECIs and GEVIs) (Lin and Schnitzer, 2016) has allowed the simultaneous recording of many cell populations involved in NVC [neurons (Urban et al., 2017), roles of astrocytes in the control of arteriole diameter, increase in local blood flow (Takano et al., 2006; Tran and Gordon, 2015), microglia as important regulators of blood flow during NVC (Hierro-Bujalance et al., 2018; Császár et al., 2021), pericytes control blood flow direction at capillary junctions, maintenance of capillary flow resistance and metabolic exchanges (Berthiaume et al., 2018; Gonzales et al., 2020)] and/or BBB permeability (Knowland et al., 2014). Along with cell-specific imaging, MPM can record local volumetric hemodynamic changes [blood flow and red blood cell velocity (Urban et al., 2017)] from surface pial vessels down to deep capillaries (Fan et al., 2020) with dedicated circulating fluorescent contrast agents (Miller et al., 2017). The unique combination of approaches offered by MPM makes it a tool of choice for investigating the complex interplay between cellular functions and vascular dynamics under awake conditions. Furthermore, the development of MPM allowing deeper imaging with better resolution (Miller et al., 2017; Wang et al., 2018) together with new faster and brighter GECIs (Dana et al., 2019; Inoue et al., 2019) to improve imaging capabilities in the mouse brain now allows to address the neurovascular components in the entire depth of the cortex, the white matter (Li B. et al., 2020) and down to the dorsal layers of the hippocampus (Ouzounov et al., 2017).

To better investigate the NVU of deeper brain areas, Miniscopes offer the opportunity to image up to 1,000 cells (Ziv et al., 2013) from distinct populations at single-cell resolution in freely behaving animals (Aharoni and Hoogland, 2019) using GECIs or GEVIs. Miniscope designs afford wide-field imaging of the cortical surface that can be extended to the entire cortical column by the means of microprism allowing for recording of both cell assembly and hemodynamics (Chia and Levene, 2009; Andermann et al., 2013; Low et al., 2014; Gulati et al., 2017) to various deep brain areas [e.g., hippocampus (Ziv et al., 2013), striatum (de Groot et al., 2020), hypothalamus (Resendez et al., 2016)] via gradient-index (GRIN) lens and relay. For example, using two GRIN relays Barretto et al. (2011) chronically monitored the progressive distortion of the microvasculature (i.e., architecture and velocity) due to glioma angiogenesis as well as properties of hippocampal neurons. To adapt the Miniscope design for hemodynamic measurements, Senarathna et al. (2019) added intrinsic optical signal (Grinvald et al., 1986) and laser speckle contrast (Miao et al., 2011) channels, enabling imaging of cerebral blood volume and flow over wide areas. Showing the Miniscope approach can also be used for BBB studies, Barr et al. (2020) used circulating contrast agents to image cocaine-induced BBB leakage in freely moving rats. Conceivably, Miniscopes will allow for comparative NVU imaging in deep regions of the brain, however hemodynamic recordings and BBB permeability are poorly addressed (often excluded from analysis) using these tools while so far limited to superficial layers of the cortex.

In contrast to the limited brain-wide capabilities of MPM and Miniscope, the MFP modality enables large-scale investigations through simultaneous recording of tens of cortical and subcortical regions of the mouse brain under freely-moving conditions (Pisano et al., 2019; Sych et al., 2019). As with MPM and Miniscope, MFP requires genetically encoded biosensors to collect changes in neuronal (Wang Y. et al., 2021) and astrocyte activity (Paukert et al., 2014; Schlegel et al., 2018) and supports simultaneous dual-color recordings that allows for combined recordings of different cell types (Meng et al., 2018). Despite its sensitivity and brain-wide coverage, MFP remains constrained to bulk changes and lacks single-cell resolution making investigations into specific non-neuronal cell population activity, hemodynamics [limited to O_2_ saturation measurement (Yu et al., 2020)] and BBB function poorly accessible so far.

From a hemodynamic point-of-view, functional ultrasound imaging (fUSI) can be considered as an alternative to fMRI by monitoring blood velocity and cerebral blood volume changes in the entire brain depth at high spatiotemporal resolution (Mace et al., 2011). Three major developments have broadened the fUSI application to neuroscience: (i) ultrasound transducer miniaturization allowed freely-behaving investigation (Urban et al., 2015), (ii) ultrasound transducer motorization achieved brain-wide coverage therefore extending the detection of activity to ~250 brain regions (Mace et al., 2018), and (iii) brain-wide volumetric imaging of behaving rodents by the mean of matrix transducer (Brunner et al., 2020). Similarly to optical modalities discussed above, several strategies have been proposed to reduce the ultrasound attenuation of the skull from transcranial (Tiran et al., 2017), thinned skull (Urban et al., 2014) to stabilized cranial window suited for awake imaging (Mace et al., 2018; Brunner et al., 2020). While fUSI enables the study of NVC regional heterogeneity (Mace et al., 2011, 2018; Sans-Dublanc et al., 2021), it has never been employed to address large scale white matter hemodynamics or BBB functions. Unlike MPM, Miniscope or MFP, fUSI cannot track cell activity via genetically-encoded indicators but can be combined with electrophysiology (Mace et al., 2018; Nunez-Elizalde et al., 2021; Sans-Dublanc et al., 2021) and cell-specific optogenetic tools (Brunner et al., 2020; Sans-Dublanc et al., 2021).

Also employed in the preclinical cerebrovascular realm, optical coherence tomography [OCT; reviewed by Yao and Wang (2014)] and optoacoustic [OA; reviewed by Ovsepian et al. (2017)] neuroimaging modalities actively support investigations on NVU/C. OCT allows for high resolution angiography of the cortical depth down to the hippocampus and including the white matter (Chong et al., 2015; Park et al., 2018). However, functional monitoring of local blood flow, velocity and O_2_ saturation (Gagnon et al., 2016) suitable under awake conditions (Li et al., 2020) are restricted to small cortical regions strongly limiting the investigation of the NVU and its heterogeneity in the entire brain. On the other hand, OA affords high resolution angiography of the cortical vascular tree but lacks spatial resolution and tissue penetration when probing mesoscale hemodynamics [i.e., hemoglobin oxygenation, O_2_ saturation (Burton et al., 2013; Tang et al., 2016)] and neural activity (Gottschalk et al., 2019). Such constraint makes OA modality limited to address the regional heterogeneity of the neurovascular unit.

Overall, imaging modalities described here range from low to high invasiveness with thinned skull or cranial window preparations (MPM and fUSI) up to insertion of device into the brain tissue (Miniscope, MFP) that may lead to structural and functional damage to the brain [i.e., tissue inflammation (Dorand et al., 2014; Bocarsly et al., 2015); brain lesions (Cole et al., 2011; Jacob et al., 2018); vessels leaking (Chia and Levene, 2009); spreading depolarization (Srienc et al., 2019)] affecting the integrity of NVU/C and inducing confounding effects in the brain cortex. These considerations further highlight the need for appropriate controls or exclusion criterion in studies using these approaches.

Beyond the established methods discussed above, the potential of combining different modalities is huge and can provide crucial information to better understand the heterogeneity and complexity driving the NVU/C at the whole brain scale. For example, scRNAseq has been performed after combined MPM and electrophysiological recordings highlighting the functional pattern and the transcriptional profile of tagged cells (Liu et al., 2020) paving the way to a better characterization of the NVU components and functions. The versatility and limited invasiveness of MFP modality allow for combination with fMRI (Schulz et al., 2012; Schlegel et al., 2018) suited for hemodynamic recordings and BBB investigations. Furthermore, the dual MFP/fUSI monitoring could provide very helpful information on the NVU heterogeneity as they generate very complementary signals (i.e., cellular and vascular) at the brain-wide scale. Similarly, Lake et al. (2020) simultaneously recorded wide-field calcium imaging with fMRI to investigate cortex-wide cell-specific activity with whole-brain hemodynamics. Moreover, Boido et al. (2019) compared the vascular and neuronal signals captured with MPM with hemodynamic changes monitored with fUSI and fMRI in the same animal.

While these studies were mostly focused on neuronal activity, the diversity of cell-specific GECI/GEVIs, optogenetic constructs and transgenic animals now available combined with the methods discussed above should support the investigation of the vascular and cellular components of the NVU at the brain-wide scale in awake rodents.

## Concluding Remarks

It is now well-accepted that the NVU displays a high degree of heterogeneity, encompassing diverse cell types and varying its composition among vessel types and anatomical regions. Recent data highlighted here is likely only a hint of how the NVU exhibits specialized function to adapt to the varied environments of the brain. Intense research on the vascular landscape of areas like the choroid plexus, where a barrier more permeable than the BBB is critical to CSF production, will surely reveal new functions to the “classical” NVU cells (Liddelow, 2015; Lun et al., 2015; Kaur et al., 2016). These cells making up the NVU, once thought limited to ECs, astrocytes, pericytes and neurons, are also joined by “newcomers,” with perivascular fibroblasts, perivascular macrophages, resident microglia and others now believed to play an active role in cerebrovascular activity (Koizumi et al., 2019; Ross et al., 2020). With each of these cell types also displaying their own regional heterogeneity across the brain (Masuda et al., 2020), it is easy to imagine that even if the cerebrovascular compartment connects into one continuum, it may in fact harbor multiple somewhat distinct regions of specialized cellular composition and function. With techniques continually improving to provide better access to the cerebrovasculature, our vision of the NVU may move from a global “one NVU fits all” perspective, to a more nuanced one where the NVU is not only metabolically serving a brain region, but participating in its specialization.

## Author Contributions

All authors listed have made a substantial, direct and intellectual contribution to the work, and approved it for publication.

## Conflict of Interest

The authors declare that the research was conducted in the absence of any commercial or financial relationships that could be construed as a potential conflict of interest.
